# Interplay between H1N1 influenza a virus infection, extracellular and intracellular respiratory tract pH, and host responses in a mouse model

**DOI:** 10.1371/journal.pone.0251473

**Published:** 2021-05-12

**Authors:** Faten A. Okda, S. Scott Perry, Richard J. Webby, Charles J. Russell

**Affiliations:** 1 Department of Infectious Diseases, St. Jude Children’s Research Hospital, Memphis, Tennessee, United States of America; 2 Veterinary Medicine Division, National Research Center, Dokki, Egypt; 3 Flow Cytometry and Cell Sorting Shared Resource, St. Jude Children’s Research Hospital, Memphis, Tennessee, United States of America; 4 Department of Microbiology, Immunology & Biochemistry, College of Medicine, The University of Tennessee Health Science Center, Memphis, Tennessee, United States of America; University of Georgia, UNITED STATES

## Abstract

During influenza A virus (IAV) entry, the hemagglutinin (HA) protein is triggered by endosomal low pH to undergo irreversible structural changes that mediate membrane fusion. HA proteins from different isolates vary in the pH at which they become activated in endosomes or become irreversible inactivated if exposed to extracellular acid. Little is known about extracellular pH in the upper respiratory tracts of mammals, how pH may shift during IAV infection, and its impact on replication of viruses that vary in HA activation pH. Here, we inoculated DBA/2J mice intranasally with A/TN/1-560/2009 (H1N1) (activation pH 5.5) or a mutant containing the destabilizing mutation HA1-Y17H (pH 6.0). We measured the kinetics of extracellular pH during infection using an optical pH-sensitive microsensor probe placed in the naris, nasal sinus, soft palate, and trachea. We also measured intracellular pH of single-cell suspensions of live, primary lung epithelial cells with various wavelength pH-sensitive dyes localized to cell membranes, cytosol, endosomes, secretory vesicles, microtubules, and lysosomes. Infection with either virus decreased extracellular pH and increased intracellular pH. Peak host immune responses were observed at 2 days post infection (DPI) and peak pH changes at 5 DPI. Extracellular and intracellular pH returned to baseline by 7 DPI in mice infected with HA1-Y17H and was restored later in wildtype-infected. Overall, IAV infection altered respiratory tract pH, which in turn modulated replication efficiency. This suggests a virus-host pH feedback loop that may select for IAV strains containing HA proteins of optimal pH stability, which may be approximately pH 5.5 in mice but may differ in other species.

## Introduction

IAVs are negative-strand RNA viruses that exhibit rapid mutations [1] and are a high risk for zoonotic infections perpetuated by host species jumping [2]. Human pandemic risk of emerging IAV strains depends on the likelihood of human-to-human transmission and the degree of virulence [3]. Independent selective pressures depend primarily upon evolutionary stimuli, novel host environments [4], and responses to preexisting immunity. These, in turn, may lead to antigenic drift [5] and antiviral drug resistance [6]. Several virological traits have been identified that support adaptation of IAVs to humans including hemagglutinin (HA) binding to alpha-2,6-linked sialic acid and HA stabilization [7,8]. Numerous studies have described the species-specific tissue distribution of sialic acid receptor isoforms in the mammalian respiratory tract; however, little is known about the pH of the mammalian respiratory tract and its contribution IAV interspecies adaptation.

HA glycoprotein trimers exhibit acid-dependent conformational changes that expose their fusion peptide regions and form proteinaceous hairpin nanomachines that promote fusion of the viral envelope with the endosomal membranes [9–12]. The pH at which HA proteins are triggered to undergo conformational changes varies by isolates but usually is within a pH range of 4.8–6.2 [7,13,14]. During entry, vesicles containing IAVs first move slowly in the cell periphery by actin-dependent active transport and then rapidly exhibit unidirectional movement toward the nucleus, where the virus-containing endocytic compartment exits the early endosome. The endosome further matures by acidifying its extracellular pH level to that of the late endosomal stage (pH ~5). A lowering of endosomal pH also stimulates M2 ion channels, leading to proton influx in the interior of the virion along with M1 dissociation from viral ribonucleoproteins, which are subsequently released into the cytosol [15–17]. The IAV genome and polymerase complexes then translocate into the nucleus where transcription and replication may occur, after which newly assembled genomes traffick via recycling endosomes via the Rab11-dependent vesicular transport pathway back to the cell periphery where they are positioned for virus assembly [18].

Two intracellular motor proteins transport IAV vesicles after endocytosis. Microfilaments carry the vesicles from the cell periphery to the perinuclear region, which are then transported by microtubules for genome release through the cytosol to reach the perinuclear area [19]. This is followed by uncoating and release of genomic RNA close to the nucleus [11,20]. HA-mediated membrane fusion for 20th Century H1N1 and H3N2 reference strains (that have relatively low HA activation pH values of 5.0–5.2) is thought to occur in the perinuclear area.

During IAV infection, intracellular pH controls different cellular processes [21]. Moreover, formation of mature viral particles requires Rab11, which mediates IAV synthesis and budding [22], and the viral M2 protein [15]. Disrupted vesicular trafficking by the ionophore monensin alters influenza RNA trafficking [19]. Intracellular pH can increase in response to growth factors from GO to G1 and into the S phase of the cell cycle [23–26]. Furthermore, chemotaxis, phagocytosis, and production of superoxide radicals reduce neutrophilic pH by 0.1–0.3 units [27,28]. Mechanisms to regulate plasma membrane (PM)–linked pH have been proposed to control ligand internalization [29]. Viral proteins interact directly with the PM during viral entry, egress during post-translational processing, and may be limited in alkaline conditions, which disrupt the cellular systems necessary for post-translational cleavage of viral proteins [21].

Recently, a stabilized HA protein has been shown to be necessary for pandemic potential in humans [30] and airborne transmission in ferrets [30–34]. Avian-adapted IAVs tend to contain HA proteins that are relatively unstable (usually activated at pH 5.5–6.2) and HA proteins from human-adapted IAVs are usually more stable (typically pH 4.8–5.5) [7,13,14]. Early pandemic H1N1 (pH1N1) isolates from 2009 had HA proteins activated at pH 5.5 [14,30], and later strains after adaptation to humans had lower HA activation pH [35–37]. A pH1N1 variant containing the mutation HA1-Y17H in the stalk region was shown to be activated at pH 6.0 [30]. This variant had loss-of-function for airborne transmission between ferrets [30], or from swine to ferrets [33], and was shown to be attenuated in DBA/2J mice due to entry in early endosomes that was associated with increased type I interferon (IFN) responses in dendritic cells [38]. Similar pH1N1 variants with destabilized HA proteins have been shown to be attenuated in mice and cultured human airway cells [39] and to be unstable in the environment after exhalation from infected ferrets [40]. It has been hypothesized, but not addressed, that attenuation in the mammalian respiratory tract of IAVs containing destabilized HA proteins may occur because of a mildly acidic extracellular environment [7]. Moreover, attenuation may also occur because of altered host responses triggered by entry in late versus early endosomes [38,39,41]. As it was unknown how IAV infection may alter extracellular and intracellular pH, in this study we aimed to determine the kinetics of respiratory pH and host responses in pH1N1-infected mice using wildtype virus (HA activation pH 5.5) and a destabilized variant (HA1-Y17H, pH 6.0) to understand virus-host interactions related to respiratory acidification.

## Materials and methods

### Mice and viruses

Mice were housed in the AAALAC-accredited Animal Resource Center at St. Jude Children’s Research Hospital (St. Jude), maintained in a 12-h light–dark cycle, and fed ad libitum standard chow and water. Animal studies were conducted as per protocols approved by the St. Jude Use and Care of Animals. DBA/2J mice were obtained from Jackson Laboratories. Recombinant A/Tennessee/1-560/2009 (H1N1) WT and HA mutant viruses are previously described [38]. A/Tennessee/1-560/2009 (H1N1) and A/California/04/09 (H1N1) differ by five amino-acid residues in the HA1 subunit: 91, 200, 206, 276, and 323. Wild-type A/Tennessee/1-560/2009 (H1N1), A/California/04/09 (H1N1), and A/England/195/2009 (H1N1) have been reported to have HA activation pH values of 5.5, while the Y17H mutation increases the activation pH to 5.9–6.0 [14,30,39].

### Animal ethics statement

Animal experiments were conducted in an ABSL2+ facility in compliance with NIH requirements and with the Animal Welfare Act. The St. Jude Animal Care and Use Committee reviewed and approved the animal experiments (protocol number 459). All animals were housed and maintained in the St. Jude Animal Resource Center (ARC), which is fully accredited by the Association for the Assessment and Accreditation of Laboratory Animal Care, International (AAALAC-I). Intranasal inoculation of influenza viruses and extracellular pH procedures were done in mice anesthetized by isoflurane. Mice were euthanized by cervical dislocation for further analysis. To minimize pain and distress, mice were regularly monitored by laboratory personnel and a team of veterinarians. Animals exhibiting evidence of morbidity or greater than 25% loss of body weight were euthanized.

### Fiber-optic phase-detection system

A microfiber optical pH meter with a needle-type–housed pH microsensor (PreSens Precision Sensing GmbH, Regensburg, Germany) was used. The pH-1 microsystem consists of a light-emitting diode with a 470-nm excitation wavelength to excite the pH sensor, an optical fiber as a signal transducer, a photomultiplier to measure emitted light from the sensor, and a chemo-optical sensor immobilized in a solid matrix at the fiber-optic tip (140-μm diameter). The chemo-optical sensor contains an H^+^-insensitive long decay time reference luminophore and an H^+^-sensitive short decay time indicator luminophore. The indicator luminophore changes its fluorescence intensity due to dynamic quenching by H^+^. The mean decline time of the luminophores represents the ratio of the two fluorescence intensities, which allows the conversion of fluorescence intensity into a phase shift. The mean phase shift indicates the intensity of the indicator luminophore and, consequently, the H^+^ concentration (i.e., pH value).

### Calibrating the fiber-optic pH microsensor

The fiber-optic microsensor, with a tip diameter of 140 μm and a tip length of 3 mm, was calibrated with six colorless pH buffer solutions (pH 4, 5, 6, 7, 8, and 9) according to the manufacturer instructions to generate a calibration curve. The paired pH buffer value and measured phase angle of each pH buffer solution were entered into the pH Solver-v07 software program to generate a sigmoidal (Boltzmann) curve fit to yield the phase_max_, phase_min_, dpH (i.e., slope), and point of inflection (pH_0_) values.

### Sterilizing the fiber-optic pH microsensor

We independently found that using the fiber-optic microsensor in different groups of mice and in different infected and uninfected mouse tissues affected the calibration curves due to the anatomy of the respiratory tract and breathing of the animals. Because of the biohazard risk associated with pH1N1, we performed in-house hospital sterilization of the fiber-optic microsensor with low pH buffer (4) and Acrylan that did not considerably influence the calibration curves generated by the pH microsensor but adequately killed the viruses.

### Infection of mice

Six-week-old female DBA/2J mice were anesthetized with isoflurane and intranasally inoculated with IAV in 30 μL PBS. We inoculated mice with 750 PFU HA1-Y17H, 750 PFU WT, or 375,000 PFU HA1-H17H. Weight was recorded daily. To obtain intracellular pH and local immune response data, nasal, soft palate, and tracheal tissues were homogenized in PBS with 5% fetal bovine serum (FBS).

### Virus titrations

Mice were euthanized at 2-, 5-, 7-, and 10-days post infection (DPI) and nasal turbinates, soft palate, and tracheae were collected. The tissues were homogenized with a Qiagen Tissue Lyser II (30/s frequency for 30s, operated twice), then clarified by centrifugation at 8,000 rpm for 15 min at 4°C in an Eppendorf 5417R centrifuge. The infectivity of the supernatants was titrated by 50% tissue culture infective dose (TCID_50_) assays [38]. The highest dilution that tested positive for virus was recorded. The TCID_50_ values were calculated by using the method of Reed and Muench, 1938.

### Optimizing extracellular pH measurements in live mice

We first conducted a pilot experiment with 10 healthy mice given different anesthetic compounds to select the best anesthetic that does not interfere with homeostatic pH. We used avertin/tribromoethanol and isoflurane. Isoflurane was introduced below the false floor of an induction chamber of 1,000-mL capacity. A 4% concentration of isoflurane was adequate for inducing short-term anesthesia (chamber volume 0.2 mL/L). The pH was first measured in nostrils, and then the sensor was carefully introduced into the nasal cavity with precise localization to achieve a stable pH reading and prevent bleeding and/or irritation. We then measured the soft palate pH and the tracheal pH with a tracheal catheter.

### Procedures for monitoring in vivo extracellular pH in the respiratory tracts of IAV infected and uninfected mice

Female DBA2J mice (*n* = 25) aged 4 to 6 weeks and weighing 12–15 g were obtained from Jackson Laboratories and adapted to standard vivarium conditions, with temperatures of 21°C–24°C and relative humidity of 50%–65%. Mice were fed standard chow and drank only autoclaved tap water. We divided the mice into three groups of five and intranasally inoculated them with 30 μL of the acid-stable 2009 pandemic H1N1 virus (WT 750), acid-destabilized H1N1 virus (HA1-Y17H 750 or HA1-Y17H 375K), or PBS. To monitor the pH of the respiratory tract at different time points post infection, five mice were randomly selected and anesthetized with isoflurane at 2, 5, 7, and 10 DPI and euthanized by cervical dislocation. Salinity and temperature were measured with the pH microsensor. Tissues were immediately collected into sterile microtubes for further analysis of the intracellular pH and local immune response by flow cytometry.

### Single-cell preparations of primary mouse nasal, soft palate, and trachea epithelial cells

The procedure was performed as previously described [42,43] with some modifications. After measuring the extracellular pH at each time point, the mice were euthanized by cervical dislocation after isoflurane exposure. In a lamellar tissue culture hood, the nasal cavity was removed with sterile surgical scissors and a scalpel. A suture loop was placed around the front incisors to immobilize the head, and the soft palate was extracted. The skin around the trachea area was removed and attached fat and salivary glands were removed. The tissues were immediately placed in small 10-cm dishes containing collecting medium (PBS with 5% FBS) on ice. Each tissue was then placed into 50 mL conical tubes containing 30 mL PBS supplemented with 5% FBS and antibiotics, on ice. The tissues were then transferred to sterile 10-cm dishes containing 10 mL PBS with 5% FBS to dissect the connective tissues with sterile forceps and surgical scissors. The tissues were washed twice and transferred to 50-mL tubes containing 10 mL 0.15% pronase and incubated overnight at 4°C. The tissues were then removed from the pronase solution, washed twice, and centrifuged at 1,400 rpm for 10 min at 4°C. Cell pellets were gently resuspended in 1 mL DNAse solution (100–200 μL/tissue), incubated for 5 min on ice, and centrifuged at 1,400 rpm (390*g*) for 5 min at 4°C. The supernatants were discarded, and the cell pellets for each tissue were resuspended in PBS with 5% FBS. The cell suspensions were plated on Primaria Plates (Becton, Dickinson & Company, Franklin Lakes, NJ) and incubated at 37°C in an atmosphere of 95% air and 5% CO_2_ for 5 h to negatively select fibroblasts. The cell suspensions were then collected from the plates and rinsed twice with 4 mL PBS containing 10% FBS. The suspension was filtered through a 40 μm strainer, followed by cell counting with trypan blue vital staining. The cells were then stained with pH dyes and subjected to fluorescence-activated cell sorting (FACS) immediately after.

### Intracellular pH measurements and local immune responses

To measure intracellular pH, pH-sensitive dyes of various excitation and emission wavelengths and an Intracellular pH Calibration Buffer kit (P35379, Thermo Fisher Scientific, Waltham, MA) were used according to the manufacturer instructions. The pH of the cytosol, microtubules, cell membranes, and lysozymes were measured with the following reagents from Thermo Fisher Scientific: pHrodo Red AM (P35372), paclitaxel Oregon Green 488 (P22310), Oregon Green 488 1,2-dihexadecanoyl-sn-glycerol-3-phosphoethanolamine (O12650), and LysoSensor Blue (L7533), respectively. Live Cell Imaging Solution (LCIS, A14291DJ, Thermo Fisher Scientific) was used to quantify the intracellular pH, and pHrodo Green and Red amine-reactive reagents (P35369 and P36600) were used to label specific antibodies for early endosome, late endosome, and secretory vesicles.

### Labeling antibodies with pHrodo green and red amine-reactive reagents

The pHrodo Red amine-reactive ester was conjugated to the EE1 antibody as a marker of early endosomes. The pHrodo Green amine-reactive ester was conjugated to RAB7 as a marker of late endosomes. The pHrodo Green amine-reactive ester was conjugated with a monoclonal anti-GRP94 antibody. GRP94 is a resident protein of the endoplasmic reticulum that is induced by accumulation of unfolded proteins and associates transiently with various newly synthesized secretory and membrane proteins. The pHrodo Red amine-reactive ester was conjugated with a monoclonal anti-CDF4 antibody (golgin 97 mon 3h811o) and an anti-mucin antibody (5ac) as Golgi markers. The antibodies were purified before labeling by dialysis with PBS (pH 7.2–7.5) to remove low molecular weight components (i.e., desalting). Labeling was then performed according to the manufacturer instructions then purified by extensive dialysis. The relative efficiency of the labeling reactions for each antibody was determined by measuring the absorbance of the dyes at their excitation maximum and protein absorbance at 280 nm.

### Optimizing intracellular pH and local immune response measurements by flow cytometry

Approximately 2 × 10^6^ cells isolated from each tissue were centrifuged at 550*g* at 4°C for 8 min, followed by resuspension of the cells in 1 mL of Sort Buffer (PBS with 5% FBS) and preparation of 10^7^ cells/mL. Three independent cell staining experiments with various combinations of antibodies, pH markers, and isotype controls were performed, and all samples were incubated for at least 30 min at 4°C and washed twice with cold PBS before analysis. To perform gating for live cells for each tissue, common antibodies, such as anti-CD4 and anti-CD8, and 4′,6-diamidino-2-phenylindole (DAPI) were used. The FACS strategy for isolating viable cells from each tissue was first determined with a FACSCanto II flow cytometer (BD Biosciences). Light scatter and DAPI was used to identify cell size–based events. Local immune responses were measured [42] after optimization and determining the appropriate gates. DAPI was not used in further tests because of its effect on cellular pH. For intracellular pH staining, ∼200,000–500,000 cells were stained, as per the manufacturer instructions. Briefly, after adding 10 μL of a pH dye to 100 μL of PowerLoad Concentrate (Thermo Fisher Scientific), the dye solution was diluted with 10 mL of LCIS and incubated at 37°C for 30 min. The cells were then washed with LCIS if needed and analyzed by using fluorescence correlation spectroscopy with an excitation/emission of 560/585 nm.

### Calibration of intracellular pH

An Intracellular pH Calibration Buffer kit contains four buffers with pH values of 4.5, 5.5, 6.5, and 7.5. The mean of three data points was plotted, and a linear trend line was fitted to obtain the pH standard curve errors. The percentage of the mean fluorescence intensity relative to three different pH buffers was used to create a linear regression to obtain the intracellular pH. Higher fluorescence intensity levels indicated lower pH values [44]. The pH values of each compartment were obtained by interpolation with the GraphPad Prism software, v9 (GraphPad, San Diego, CA).

### Local immune responses

The local immune responses of mice infected with WT or Y17H IAVs were measured and compared with those of PBS-treated mice [45]. Briefly, approximately 2 × 10^6^ cells per tissue from each treatment group at different time points were stained with a Live/Dead viability dye (Life Technologies) according to manufacturer instructions. The cells were incubated in blocking solution containing 5% FBS and 1% FcBlock (eBiosciences, San Diego, CA) in PBS and stained with immunophenotyping antibodies against CD11a, CD11b, F4/80, CD4, and CD8 for 30 min at room temperature. The cells were then washed and fixed with 0.4% paraformaldehyde in PBS. Data were acquired with a BD LSRII flow cytometer and BD FACSDiva software (BD Bioscience).

### Statistical analysis

Measurements were repeated three times for each tissue and animal. Data were exported via the instrument software and evaluated with GraphPad Prism, v9.0. Statistical comparisons were made with two-way ANOVAs and appropriate posthoc analyses.

## Results

### Calibration of the fiber-optic pH microsensor

We calibrated eight fiber-optic pH microsensors and measured the acidity of several pH buffers by using a pH electrode with a linear correlation in the pH range of 4–9 between the measured and expected pH values.

**Fig 1:**
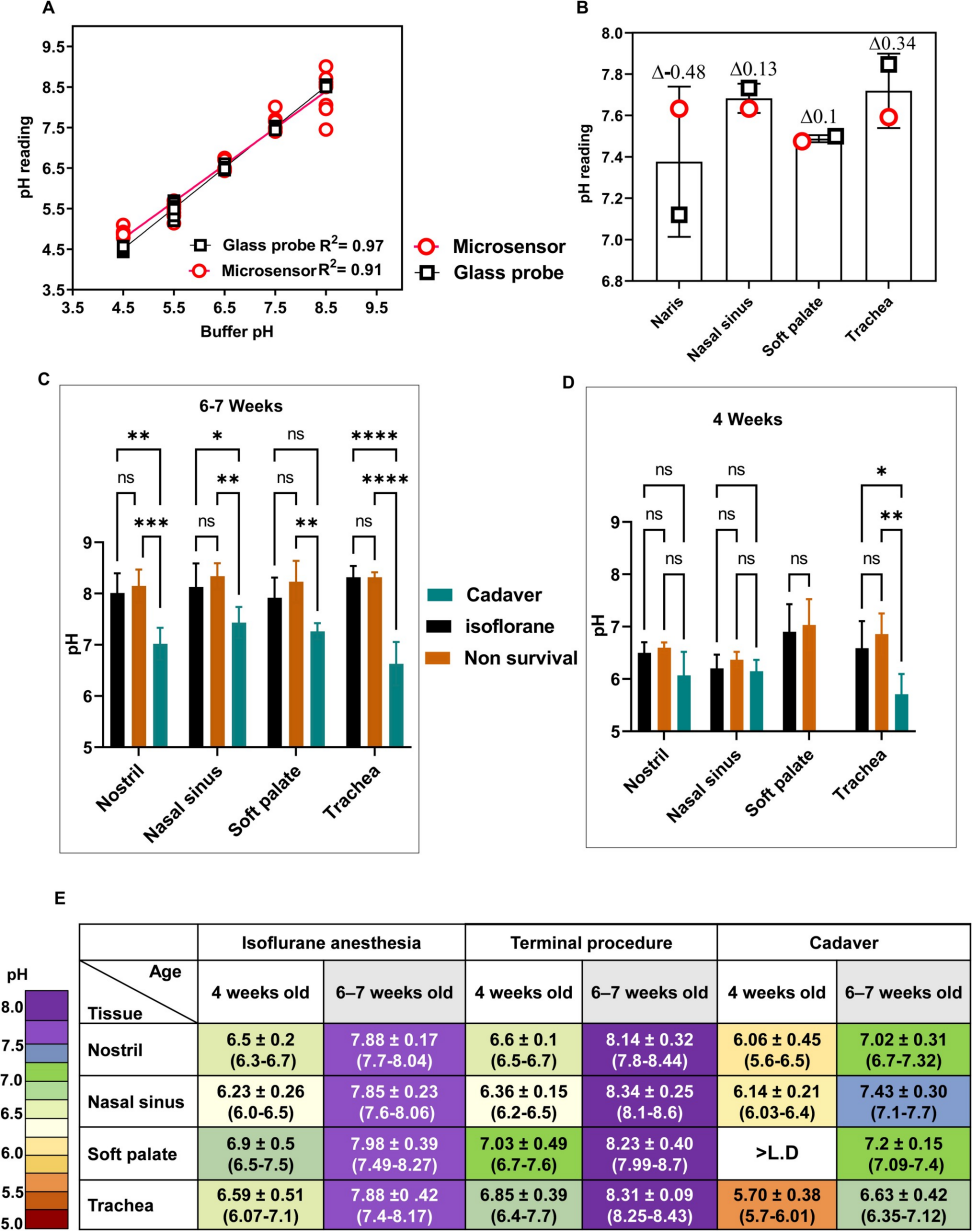
Calibration and optimization of microsensor-measured pH in the respiratory tracts of uninfected DBA/2 mice. (A) Calibration curves of pH-adjusted buffers using both a glass electrode probe (black squares) and a microsensor (red circles). Values were measured at 25°C with both probes in the same buffer simultaneously. The number of readings was recorded at each pH, and individual data points are shown. Data were fitted by least squares regression and *R*^*2*^ values are shown. (B) Respiratory pH readings in uninfected DBA/2 mice under isoflurane-induced anesthesia. In three independent experiments, pH values were measured for groups of three mice with a pH microsensor (red circles) or a glass probe (black squares). The differences in pH values between the calibrated glass electrode and pH microsensor are reported above each column. (C and D) Extracellular respiratory pH values of uninfected DBA/2 mice that were 4 weeks old (C) or 6–7 weeks old (D). In two independent experiments, groups of three mice under isoflurane-induced anesthesia were used to measure respiratory pH in nares, nasal sinuses, soft palate, and tracheae with the pH microsensor probe. The value for soft palate cadaver for 4-week-old mice was less than the limit of detection. Values are the mean (±SD), with significance indicated as follows: *, *P <* 0.05; **, *P <* 0.01; ***, *P <* 0.001; ****, *P <* 0.0001. (E) Murine extracellular respiratory tract pH values in uninfected DBA/2 mice. Values are given for groups of three mice at two different ages, 4 weeks old and 6–7 weeks. Values are the range and combined mean (±SD) from two experiments for each age group under three conditions: Isoflurane-induced anesthesia (*n* = 12), terminal procedure in which the mice were euthanized directly after measurements (*n* = 12), and mice euthanized by cervical dislocation (Cadaver) (*n* = 12). Two way-ANOVA and Tukey posthoc tests were performed.

Our calibration experiments had an R^2^ = 0.91, which was congruent with greatest accuracy in the pH range of 5.5–7.5 (Fig 1A) compared to the pH range using thin glass pH electrode (Fig 1B).

### Optimizing extracellular pH measurements in uninfected mice

We first determined the best anesthetic reagents to preclude interference with homeostatic pH in mice aged 4 weeks and 6–7 weeks. Isoflurane resulted in the least interference in pH measurements. The mean pH in 4-week-old mice was 6.5 in the nostrils, 6.2 in the nasal sinuses, 6.9 in the soft palate, and 6.6 in the tracheae (Fig 1C and 1E). The mean pH in the 6–7-week-old mice was 7.88 in the nostrils, 7.85 in the nasal sinuses, 7.98 in the soft palate, and 7.88 in the tracheae (Fig 1D and 1E). In summary, young uninfected mice had slightly more acidic upper respiratory tracts than did older mice.

### Viral titers

**Fig 2:**
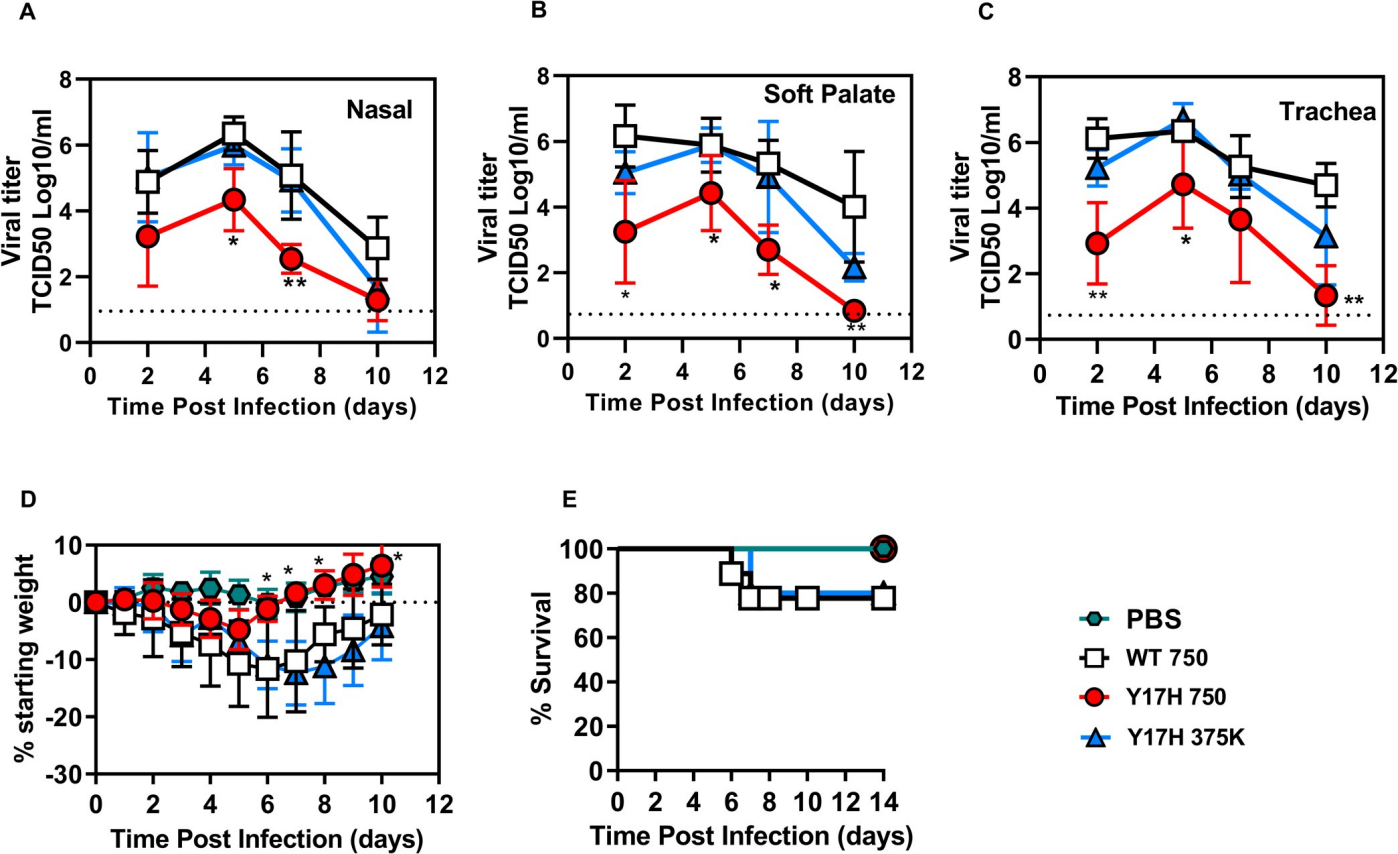
Tissue viral titers and body weight loss in DBA/2 mice infected with A/TN/09 variants. (A-C) Nasal, soft palate, and tracheal tissue titers at 2, 5, 7- and 10-days post infection (DPI). The inoculated doses were 750 PFU for WT and 750 or 375,000 PFU for Y17H. Values are the mean (±SD) for three mice, with a total of 12 mice at each time point. (D-E) Changes from starting body weight and percent survival of DBA/2 mice after inoculation with 750 PFU of WT and 750 or 375000 PFU of Y17H. Symbols used in all panels as follows: PBS (green hexagon), WT 750 (black squares), Y17H 750 (red circles), and Y17H 375 (blue triangles). Statistical significance was determined by two-way ANOVA and Tukey posthoc analysis, comparing each set of results to those for WT virus. Significant statistical differences are indicated as follows: *, *P <* 0.05; **, *P <* 0.01; ***, *P <* 0.001; ****, *P <* 0.0001.

We inoculated groups of DBA/2J mice with 750 PFU of WT or HA1-Y17H. To normalize the viral titers and body weight loss for subsequent studies of host responses, we included an additional group of mice infected with 375,000 PFU Y17H as we had established previously[38]. Mice inoculated with Y17H 375K and WT 750 resulted in mean nasal viral titers that were higher by approximately 2 logs at 2 DPI (*P* = 0.05) and at 5 DPI (*P* = 0.007) (Fig 2A) than were those of Y17H 750 PFU–infected mice. In the soft palate, WT 750 produced a mean viral titer that was 3 logs higher than that of Y17H 750 and 1 log higher than that of Y17H 375K at 2 DPI (Fig 2B). In the trachea, WT 750 and Y17H 375K exhibited the same kinetics during infection, with viral titers of 10^6^ TCID_50_/mL at 2 and 5 DPI followed by gradual declines at 7 DPI (Fig 2C). In contrast, viral titers of Y17H 750 were lower by a factor of 3 log at 2 DPI and by 20-fold at 5 DPI. Infection in the trachea of Y17H 375K yielded similar titers to WT 750 except for reduced titers in nasal, soft palate, and tracheal tissues that were approximately 0- to 0.5-log lower at 2 DPI (*P* = 0.017) and at 5 DPI (*P* = 0.01). Overall, the HA-destabilizing Y17H substitution was highly attenuating but could be partially overcome by inoculation with a 500-fold higher dose.

### Weight loss and survival

The WT 750 and Y17H 375K groups had maximum body weight losses of approximately 20%, whereas the Y17H 750 group has significantly less (*P* < 0.05 at 7–10 DPI) (Fig 2D). Thus, Y17H 750 did not induce pathogenicity such as that caused by Y17H 375K of WT 750. Overall, these data indicate that increasing the dose of Y17H IAV to 375,000 PFU caused similar weight loss and comparable (albeit unequal) nasal, soft palate, and tracheal viral titers with those of WT 750.

### Extracellular pH measurements in uninfected and infected mice

**Fig 3:**
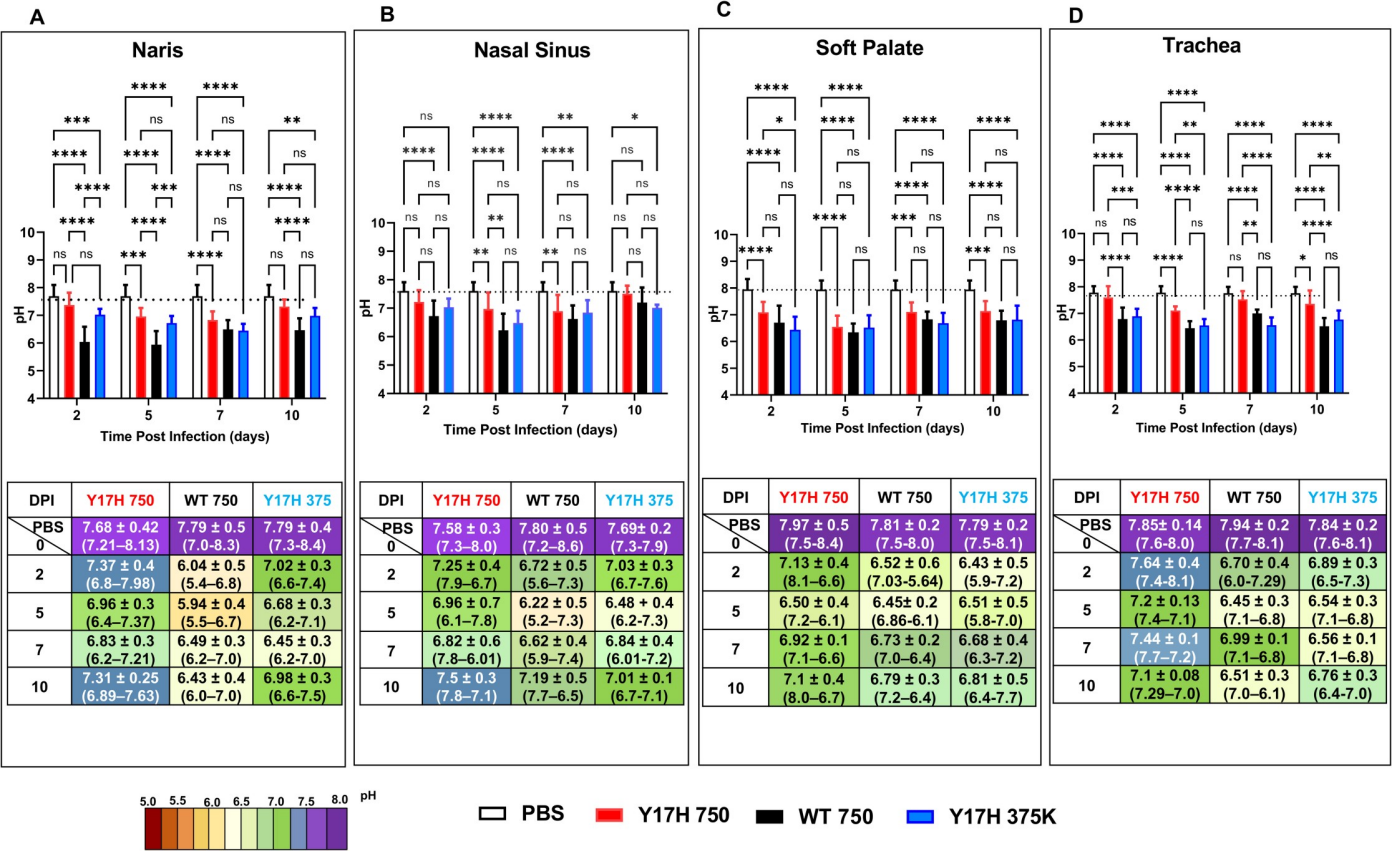
Extracellular respiratory tract pH in DBA/2 mice after infection. Mice were intranasally inoculated with PBS, WT virus (750 PFU), or Y17H virus (750 or 375,000 PFU). **(A-D)** Naris pH (A), nasal cavity pH (B), soft palate pH (C), and tracheal pH (D). Reported values are the means ± SD (*n* =  10). The associated tables under the bar graphs contain the means ± SD (*n*  =  10), and the ranges are indicated in parentheses. Values are the combined mean (±SD) from two independent experiments with 40 mice total. Mice were intranasally inoculated with PBS, 750 PFU of WT virus, 750 PFU of Y17H virus, or 375,000 PFU of Y17H virus. After 2, 5, 7, or 10 days of infection, extracellular pH values were measured in the nares, nasal cavity, soft palate, and trachea with an optical pH microsensor. Measurements were taken under isoflurane-induced anesthesia. The mice were then euthanized, and tissues were collected and processed for further analysis. Two way-ANOVA and Tukey posthoc tests were performed on each day. Significant statistical differences are indicated as follows: *, *P <* 0.05; **, *P <* 0.01; ***, *P <* 0.001; ****, *P <* 0.0001. Statistical significance tests compared the results to those for PBS-treated are shown in purple (*) or in connected lines and black (*) between groups. The bars for each group are colored as follows: PBS (white with black outline), WT (solid black), Y17H750-PFU (solid red), and Y17H 375-PFU (solid blue).

To measure the extracellular pH in murine nasal, soft palate, and tracheal tissues, we intranasally inoculated 5-week-old mice with WT 750, Y17H 750, Y17H 375K, or PBS. In PBS-treated mice, the mean pH values ranged from 7.6–8.0 in all four tissues (Fig 3). The mean pH in the naris of infected mice at 2 and 5 DPI decreased to approximately 7.37 and 6.96, respectively (*P* < 0.0003, compared to PBS-treated mice), for Y17H 750, to 7.02 and 6.68, respectively (*P* < 0.0001 compared to PBS-treated mice), for Y17H 375K, and 6.04 and 5.94, respectively, for WT 750. At 7 DPI, mice infected with Y17H 750 had a naris pH of 6.89, which recovered to approximately 7.31 by 10 DPI. In contrast, at 7 DPI WT 750 and Y17H 375K inoculation resulted in a naris pH of approximately 6.5 and was not restored to the naris uninfected pH by 10 DPI (Fig 3A).

Uninfected nasal sinus pH was approximately 7.7. Infection by Y17H 750 reduced nasal sinus pH to 7.25 at 2 DPI, 6.96 at 5 DPI, and 6.82 at 7 DPI before recovery to 7.5 at 10 DPI (Fig 3B). Infection with WT 750 and Y17H 375K reduced nasal sinus pH even lower (to 6.22 and 6.48, respectively) at 5 DPI and did not fully recover by 10 DPI.

Uninfected soft palate pH was approximately 7.9. The mean pH at 2 DPI was reduced to 7.1, 6.5, and 6.4 for Y17H 750, WT 750, and Y17H 375K, respectively (Fig 3C). However, at 5 DPI, all three IAVs had mean soft palate pH values of approximately 6.5. At 7 DPI, the three IAVs increased the pH to 6.9 for Y17H 750, 6.7 for WT 750, and 6.6 for Y17H 375K. The mean pH returned to neutral with Y17H 750 at 10 DPI, whereas WT 750 and Y17H 375K maintained mildly acidic.

Uninfected tracheal pH was approximately 7.9. Tracheal pH decreased, but not less than 7, during infection with Y17H 750 (Fig 3D). In contrast, WT 750 shifted tracheal pH to acidity during infection, with a mean pH of 6.7, 6.4, 6.9, and 6.5 at 2, 5, 7, and 10 DPI, respectively. Y17H 375K had similar kinetics of tracheal acidification. Overall, these findings demonstrate that the respiratory tract of infected DBA/2J mice is slightly acidic at 2 and 5 DPI, but not less than a pH of 6.2.

Previous studies showed that infectivity of WT virus in pH 6.4 media decayed at rate similar to neutral-pH media, while Y17H was much more rapidly inactivated when exposed to a pH of 6.4 [38]. In the present study, infection with Y17H 750 did not sufficiently acidify the respiratory tract to a value that would be expected to inactivate the virus, while infection with higher-dose Y17H 375K resulted in a respiratory acidification sufficiently low to cause extracellular inactivation. Extracellular acidification was not low enough to reach a value capable of inactivating WT.

### Salinity and temperature in infected and uninfected mice

**Fig 4:**
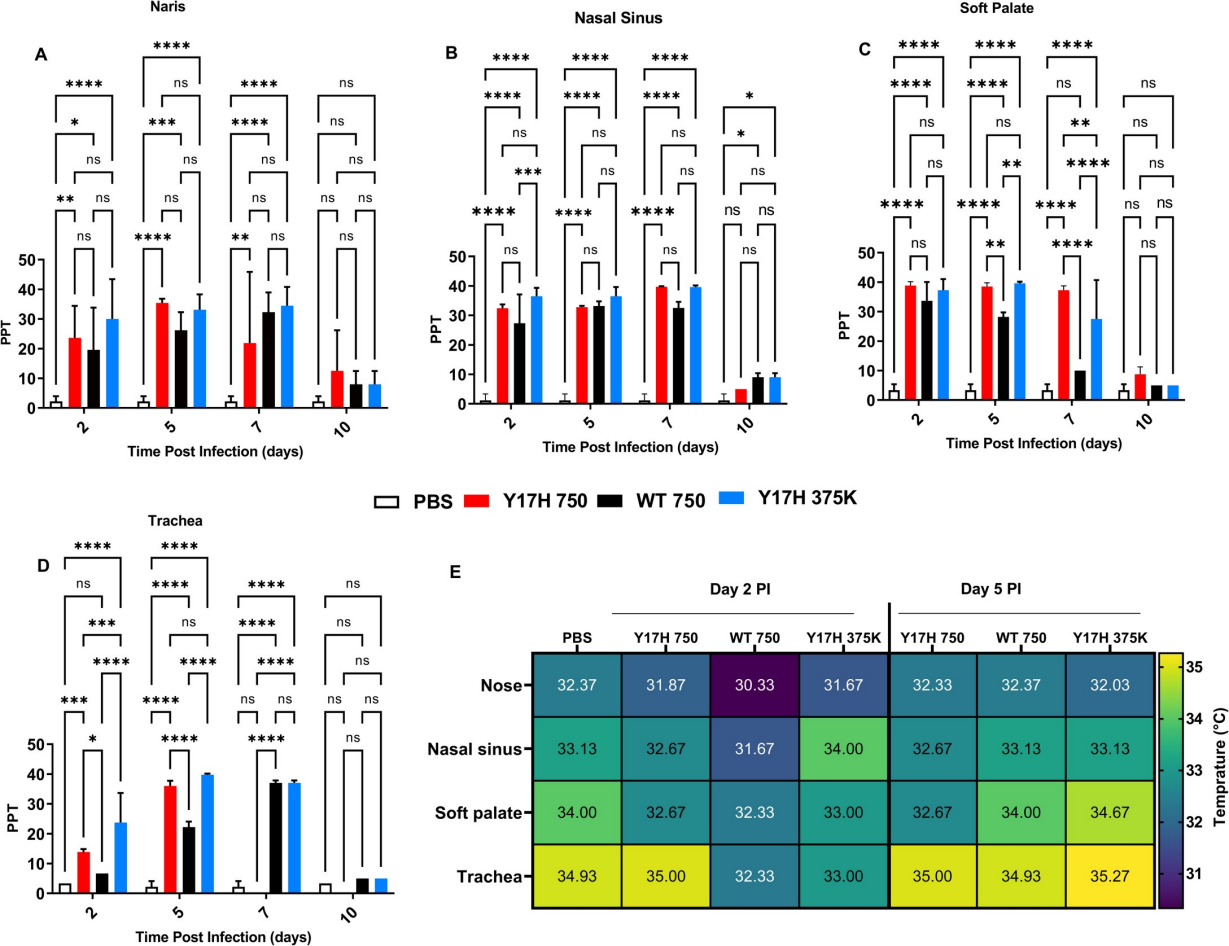
Extracellular respiratory tract salinity and temperature in DBA/2 mice after infection. Mice were intranasally inoculated with PBS, WT virus (750 PFU), or Y17H virus (750 or 375,000 PFU). **(A-D)** Values from nares, nasal cavity, soft palate, and tracheal salinity expressed as parts per thousand (PPT). After 2, 5, 7, or 10 days of infection, extracellular salinity and temperature were recorded. Reported values are means ± SD (*n*  =  10). Two way-ANOVA and Tukey posthoc tests were performed separately for each day, and significant statistical differences are indicated as follows: * P<0.5, ***P<0.01, ***P<0.001, and ****P<0.0001. (**E**) Summary of temperature values (degrees Celsius) for each group with a colored heat map (right).

In the nares, Y17H 375K resulted in the highest saline concentration at 2 DPI, in comparison with PBS, and no statistical difference with Y17H 750 or WT 750. At 5 DPI, all three IAVs significantly differed from PBS treatment (*P* < 0.0001), and WT 750 and Y17H 750 also significantly differed (*P* = 0.001). At 7 DPI, only WT 750 and Y17H 375K significantly differed from PBS (Fig 4A). In the nasal sinuses, saline concentrations at 2 DPI in both Y17H 750– and Y17H 375K–infected mice differed from that of PBS-treated mice. At 5 DPI, all three IAVs differed from PBS, but with no significant differences between each group. At 7 DPI, only WT 750 increased saline concentrations over that of PBS group (Fig 4B). In the soft palate, all three IAVs resulted in higher saline concentrations over that of PBS at 2 and 5 DPI. At 5 DPI, WT 750 had lower saline concentrations than both Y17H 750 and Y17H 375K, and at 7 DPI, only WT 750 was significantly higher than PBS (Fig 4C). In the trachea, WT 750 and Y17H 750 differed from PBS at 2 DPI, whereas Y17H 375K did not differ from PBS or WT 750 and Y17H 750. At 5 DPI, salinity increased for all IAVs, and Y17H 750 differed from WT 750. At 7 DPI, Y17H 750 salinity levels returned to baseline, which ranged from 0 to 10 parts per trillion. WT 750 and Y17H 375K salinity returned to baseline at 10 DPI (Fig 4A–4D). Overall, salinity increased from 2 DPI to 10 DPI, with a peak at 7 DPI, for all IAVs (Fig 4). At 10 DPI, all IAVs decreased saline concentration. The nasal sinuses and soft palate showed the highest increase in saline concentrations, as compared with the corresponding IAVs in the nares and tracheae.

The baseline respiratory tract temperature of DBA/2J mice was 32.37°C and 33.13°C in the nose and nasal sinuses, respectively, with higher temperatures in the soft palate (34°C) and trachea (34.93°C). At 2 and 5 DPI, the nasal and soft palate temperatures did not change, except a 1-point increase at 5 DPI with Y17H 375K. The temperature slightly increased with Y17H 750 at 2 and 5 DPI and with Y17H 375K at 5 DPI (Fig 4E). Therefore, infection with either WT or mutant IAVs did not affect respiratory tract temperature in DBA/2J mice.

### Local immune responses

**Fig 5:**
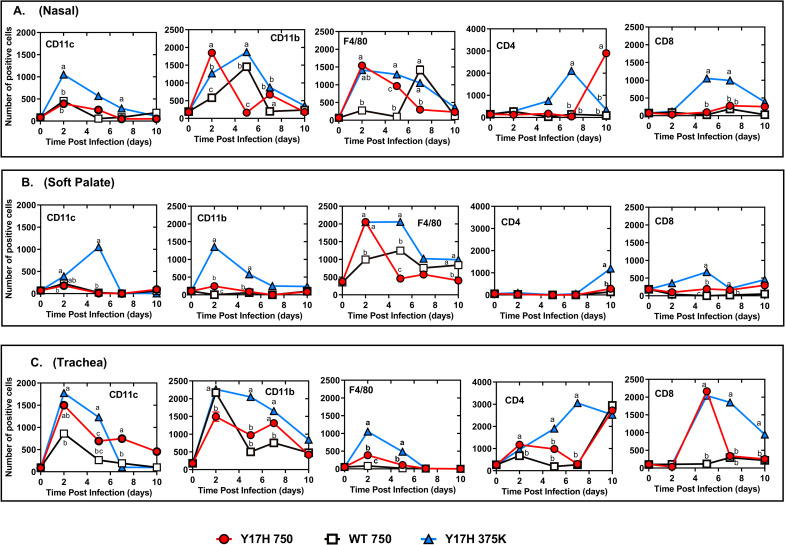
Local immune cell infiltration in the respiratory tracts of DBA/2 mice after infection. Mice were inoculated intranasally with PBS, WT virus (750 PFU), or Y17H virus (750 or 375,000 PFU). (**A-C**) Values of the mean florescence intensity of positive immune cell populations in response to viral infection in nasal tissues (A), soft palate (B), and trachea (C). The local immune cells were stained with antibodies raised against CD11c, CD11b, F4/80, CD4, and CD8 for each tissue, as labeled in the top left corner of each panel. After 2, 5, 7, or 10 days of infection, single epithelial cells from a homogenate of three mice per group were isolated, and the number of CD11c^+^, CD11b^+^, F4/80^+^, CD4^+^, and CD8^+^ cells were determined by flow cytometric staining and analyses. The displayed values are the mean (±SD) of two independent experiments, with each experiment including a homogenate of three replicates. Statistical analysis was performed by using two-way ANOVA and Tukey posthoc tests. At each time point, means are denoted by a different lower-case letter that indicate significant statistical differences between groups (*P* = 0.001), whereas means with the same letters indicate no significant statistical difference.

Despite the heterogeneity and complexity of non-lymphoid tissues, we generated single-cell suspensions for flow cytometric analysis of tissue-resident immune cells. We used a 5-fluorochrome panel for cell staining and a standardized gating strategy that simultaneously identifies and quantifies immune cell populations of macrophages, dendritic cells (DCs), and CD8^+^ and CD4^+^ T cells in the nose (Fig 5A), soft palate (Fig 5B), and trachea (Fig 5C) of infected and uninfected mice. The macrophage and DC populations gradually increased at 2 and 5 DPI and declined by 7 DPI. The population of CD4^+^ T cells notably increased with Y17H 750 and Y17H 375K at 7 DPI. DC frequency in the nose (Fig 5A) and soft palate (Fig 5B) peaked at 2 DPI in with all IAVs and rapidly declined thereafter. CD11b-expressing cells peaked at 2 DPI with Y17H 750 and Y17H 375K and then rapidly declined at 5 DPI. DCs gradually increased with Y17H 750 and Y17H 375K starting 2 DPI and peaked at 5 DPI, followed by a decline at 7 DPI. In contrast, macrophage populations peaked at 2 DPI with Y17H 750 and Y17H 375K and gradually decreased until 10 DPI. Macrophages populations peaked at 7 DPI with WT 750 and then suddenly declined at 10 DPI. The frequencies of T cells in the soft palate did not change with any treatment group. Tracheal immune responses showed similar kinetics for DC, CD11b, and CD11c populations in all treatment groups, which peaked at 2 DPI and then gradually decreased by 10 DPI. Interestingly, tracheal macrophage populations diverged from those in the nose. The macrophages in WT 750–infected mice peaked at 2 DPI and suddenly declined at 5 DPI, whereas the macrophages in Y17H 750– and Y17H 375K–infected mice increased at 2 DPI, peaking from 5 to 10 DPI. In the Y17H 750– and Y17H 375K–infected mice, T-cell populations began accumulating in the nose at 5 DPI, soft plate at 2 DPI, and trachea at 5 DPI (Fig 5C). However, T cells markedly increased until 10 DPI in both the nose (Fig 5A) and soft palate (Fig 5B) and declined in the trachea at 10 DPI (Fig 5C). The frequency of CD8^+^ T cells declined over the first 7 days with WT 750, with high accumulation at 7 DPI. CD4^+^ T cells peaked by 10 DPI in the noses of Y17H 750– and Y17H 375K–infected mice but did not increase with WT 750. CD4^+^ T cells were absent in the soft palate (Fig 5B), and tracheal CD4^+^ T cells began accumulating at 2 DPI, with a marked increase at 10 DPI with all IAVs (Fig 5C). These results indicate that pH and local innate immune responses are associated with each other during IAV infections.

### Intracellular pH in respiratory tract epithelial cells

**Fig 6:**
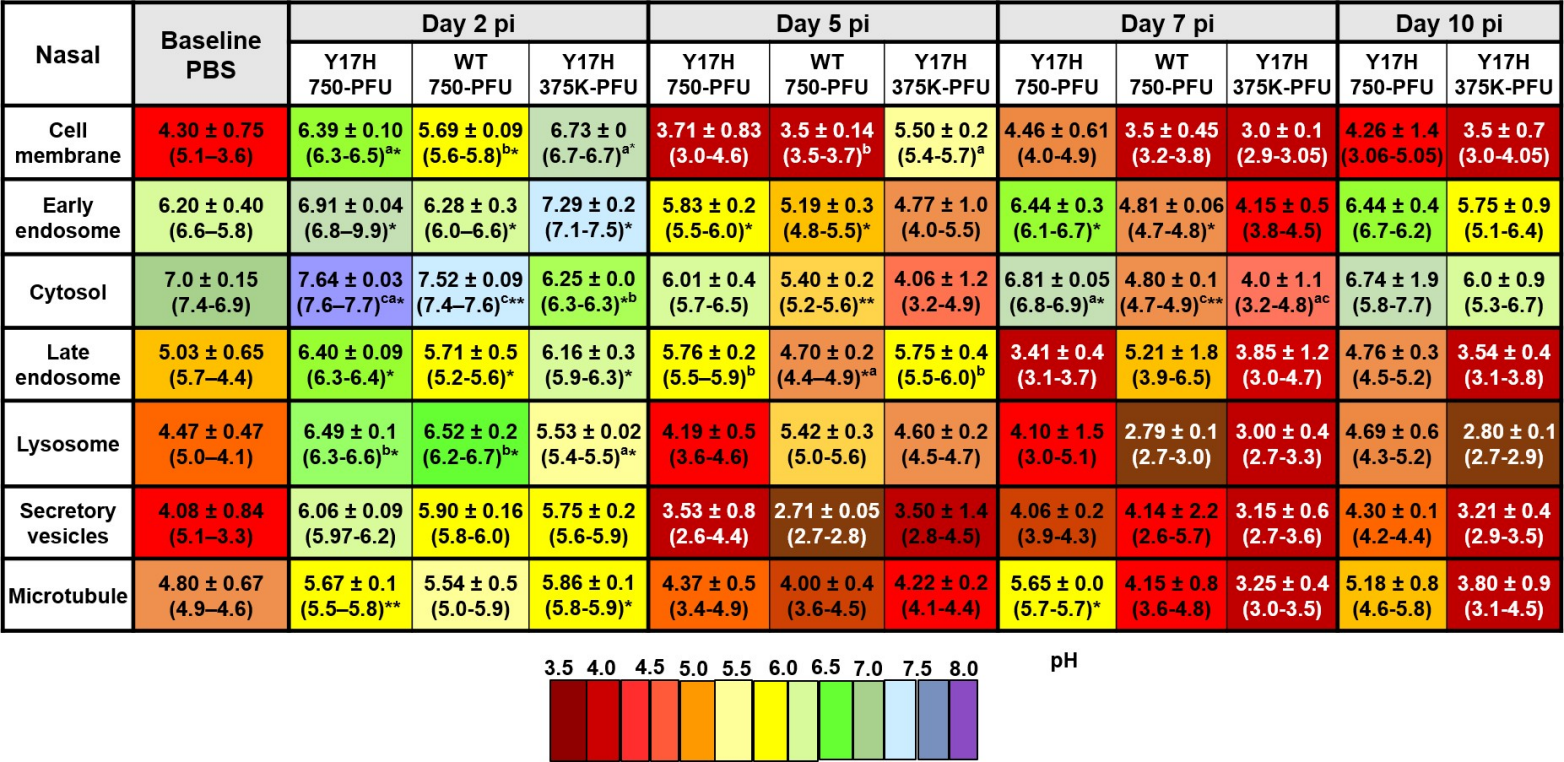
Intracellular pH values of uninfected (PBS) and infected live epithelial cells isolated from the nasal tissues of DBA/2J mice. Mice were intranasally inoculated with PBS, WT virus (750 PFU), or Y17H virus (750 or 375,000 PFU). After 2, 5, 7, or 10 days of infection, following the previously reported extracellular pH measurements, single epithelial cells from a homogenate of three mice per group were isolated and analyzed with high-throughput flow cytometric analyses. A panel of the following pH dyes with different wavelengths included Oregon Green DHPE for phospholipid cell membrane pH, pHrodo Red succinimidyle (NHW) ester conjugated with an EE1 antibody as a marker of early endosomes; pHrodo Red AM for cytosolic pH; pHrodo Green STP ester conjugated with RAB7 as a marker of late endosomes; and LysoSensor Blue as a marker of lysozomes. A pHrodo Red succinimidyle (NHW) ester conjugated with a GRP94 monoclonal antibody was used as a marker of the secretory vesicles. Paclitaxel Oregon Green 488 was used as a pH marker of microtubules. Values are mean (±SD) and ranges of the homogenates of three mice from two indepenant experiments. Data were calculated from a linear standard curve generated from an intracellular pH calibration kits for each probe with Graph Pad Prism 7 (GraphPad). After calculating all fitted data, two way-ANOVA and Tukey posthoc tests for two independent experiments were performed separately for each time point. Statistically significant differences for virus-infected groups compared to the PBS group are indicated as follows: *P < 0.5, ***P < 0.01, ***P < 0.001, and ****P < 0.0001. At each time point, statistically significant differences between the three virus-infected groups are denoted by having a different lower-case letter (a, b, or c) superscript (*P* = 0.001). Values with the same lower-case letters indicate no significant statistical difference between those groups.

**Fig 7:**
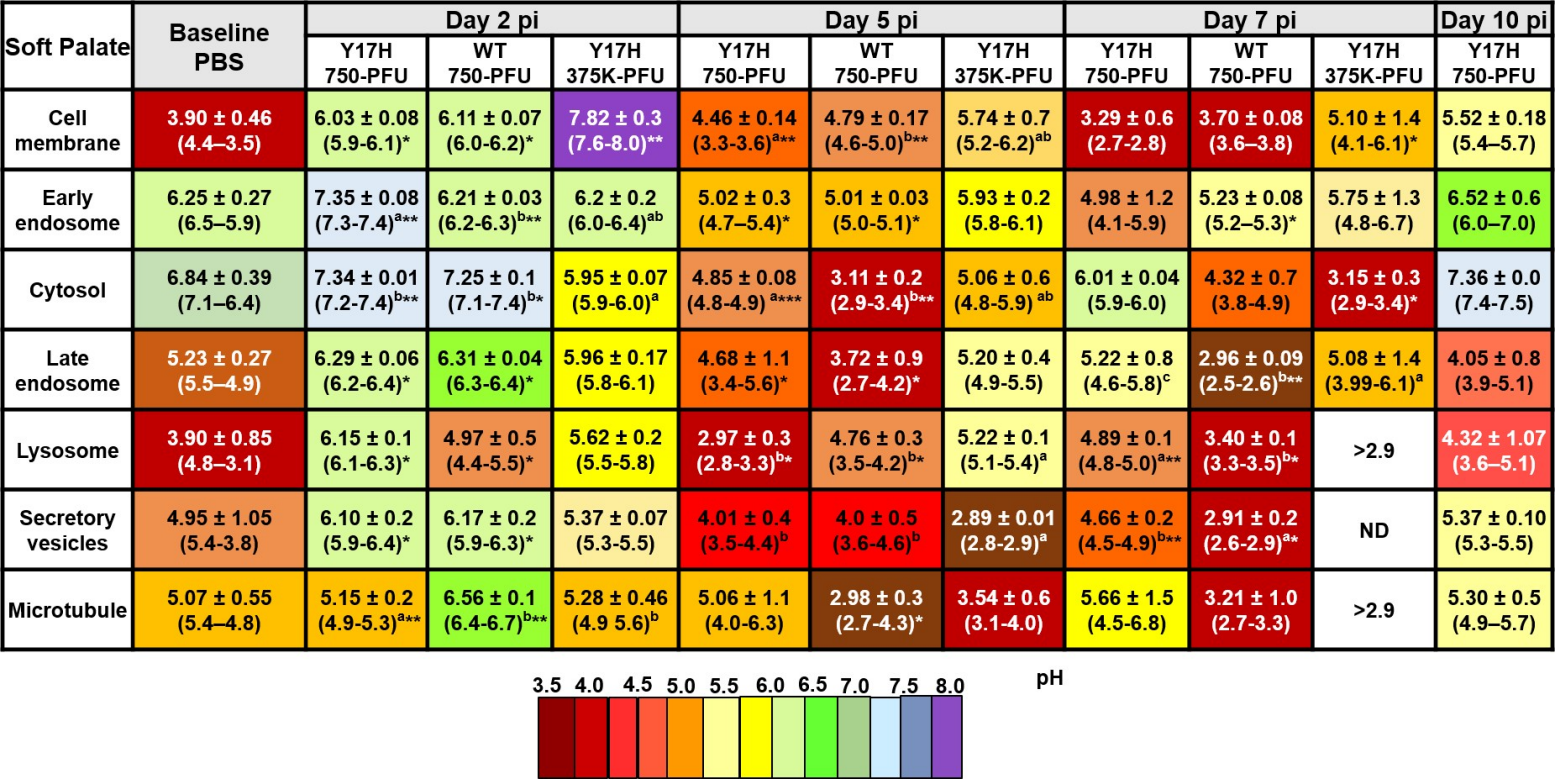
Intracellular pH values of individual uninfected and infected live epithelial cells isolated from the soft palate tissues of DBA/2J mice. Mice were intranasally inoculated, and cellular pH values were determined as described in Fig 6.

**Fig 8:**
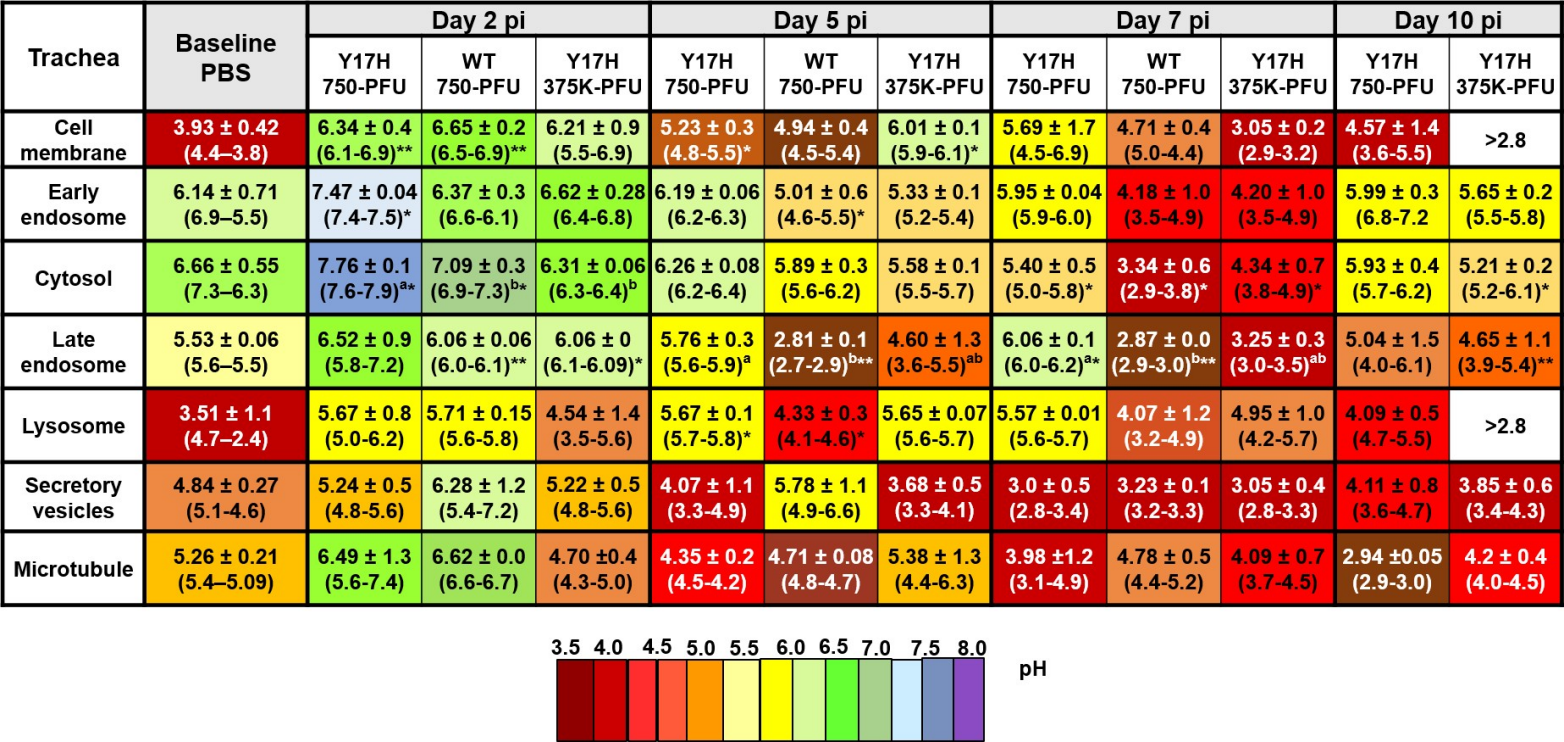
Intracellular pH values of individual uninfected and infected live epithelial cells isolated from the tracheal tissues of DBA/2J mice. Mice were intranasally inoculated, and cellular pH values were determined as described in Fig 6.

pH values of various subcellular compartments were determined by flow cytometric analysis. Each mouse yielded 2.5–3.0 × 10^6^ single live epithelial cells. Conjugated fluorescent pH indicator dyes were successfully loaded into the cells as acetoxymethyl esters, which are permeable to cell membranes, followed by cleavage inside the cell by ubiquitous intracellular esterase, which releases a charged species that cannot exit the cell. Each pH indicator was optimized according to the excitation or emission spectra, and a set of three different wavelengths were used. To measure the subcellular pH in murine nose, soft palate, and tracheal cells, we intranasally inoculated mice with WT 750, Y17H 750, Y17H 375K, or PBS. We then isolated single live cells from each group for flow cytometry analysis as mentioned above. The baseline subcellular pH was highly selective, and the phospholipid-containing cell membranes were acidic, conferring the selective permeability of the cell membrane. The cytosolic pH and early endosomal pH were more alkaline compared to other specific compartments measured, and the pH gradually decreased in the late endosome, lysozyme, secretory vesicles, and microtubules. The Golgi complex exhibited a baseline pH like that of the cytosol. The subcellular pH of nasal, soft palate, and tracheal tissues in uninfected and infected DBA/2J mice are shown in Figs 6–8, respectively.

In PBS-treated mice, the mean nasal epithelial cell membrane pH was 4.3 (Fig 6), whereas the mean soft palate and tracheal cell membrane pH values were 3.90 (Fig 7) and 3.93 (Fig 8), respectively. The cell membrane pH in the nasal epithelial cells in mice infected with all three IAVs increased significantly at 2 DPI, with mean pH values of 6.39 (*P* < 0.5), 5.69 (*P* < 0.5), and 6.73 (*P* < 0.5) for Y17H 750, WT 750, and Y17H 375K, respectively. At 5 DPI, all three groups had sharply decreased values of mean nasal cell membrane pH, as compared with the pH values at 2 DPI and baseline. WT 750 and Y17H 375 cell membrane pH remained low at 7 DPI and at 10 DPI, with a low number of live cells. In contrast, Y17H 750 cell membrane pH returned to that of baseline at 7 and 10 DPI (Fig 6). The soft palate cell membrane mean pH sharply increased at 2 DPI, with mean values of 6.3 (*P* < 0.5), 6.11 (*P* < 0.02), and 7.82 (*P* < 0.01) for mice inoculated with Y17H 750, WT 750, and Y17H 375K, respectively, as compared to that of PBS-treated mice. At 5 DPI, the cell membrane pH decreased in all groups, with significant differences between Y17H 750 and WT 750, and continuously decreased at 7 and 10 DPI. The number of live cells at 10 DPI was low, except for those isolated from Y17H 750–infected mice, which maintained high numbers of live cells and a mean pH of 5.1 (*P* = 0.02) at 7 DPI and 5.2 at 10 DPI (Fig 7). The mean pH of the tracheal epithelial cell membranes in infected mice 2 DPI increased to approximately 6.34 (*P* < 0.01), 6.5 (*P* < 0.001), and 6.21 for Y17H 750, WT 750, and Y17H 375K, respectively, as compared with that of PBS-treated mice. At 5 and 7 DPI, mice inoculated with Y17H 750 and WT 750 exhibited decreased cell membrane pH values to a mean of 5.23 at 5 DPI and 5.6 at 7 DPI for Y17H 750 and to 4.94 at 5 DPI and 4.7 at DPI for WT 750. Although the mean tracheal cell membrane pH with Y17H 375K was 6.01 at 5 DPI, it sharply declined to 3.05 at 7 DPI. At 10 DPI, the mean tracheal cell membrane pH with Y17H 750 recovered to baseline, whereas the pH with WT 750 and Y17H 375 were below the limit of detection with a low number of live cells (Fig 8).

In PBS-treated mice, the mean pH of early endosomes in the nasal epithelial was 6.20 (Fig 6), and the mean pH in the soft palate and tracheal epithelia cells were 6.25 (Fig 7) and 6.14 (Fig 8), respectively. All three groups of infected mice had increased nasal endosomal pH at 2 DPI (*P* < 0.02) to a mean of 6.91, 6.28, and 7.29, respectively, followed by decline at 5 DPI. The early endosome pH with Y17H 750 returned to a baseline pH of 6.44 (*P* < 0.5), whereas the early endosome pH with WT 750 and Y17H 375K decreased to mean values of 4.81 (*P* < 0.5) and 4.15 respectively at 7 DPI. At 10 DPI, the early endosome pH with WT 750 was below the limit of detection, whereas that with Y17H 750 remained at baseline and Y17H 375K slightly increased to 5.75 (Fig 6).

The cytosolic baseline pH was alkaline in nasal, soft palate, and tracheal tissues, with mean values of 7.0, 6.84, and 6.66, respectively. Like the pH changes in the early endosomes, the nasal cytosolic pH significantly increased at 2 DPI to 7.6 (*P* < 0.5) in Y17H (750PFU) and 7.52 in WT (750PFU) (*P* < 0.01) and slightly decreased to 6.25 (*P* < 0.5) in Y17H (375KPFU), as compared to that of PBS-treated mice. WT 750 and Y17H 375K differed from each other. The Y17H 750–infected mice had slightly decreased the mean pH (6.01) at 5 DPI followed by an increase to 6.81 (*P* < 0.01) at 7 DPI, with significantly increased mean pH values of 5.40 and 4.80 with WT 750 at 5 DPI (*P* < 0.01) and 7 DPI (*P* < 0.01), respectively. Although the cytosolic pH with Y17H 375K was less than that with Y17H 750 at 5 and 7 DPI, both groups returned to baseline pH at 10 DPI, whereas the cytosolic pH with WT 750 was below the limit of detection, with a low number of live cells (Fig 6).

The pH in the cytosol of the soft palate epithelium was like the nasal cytosolic pH at 2 and 5 DPI for all groups, except that the mean pH value with Y17H 750 was acidic (4.85, *P* < 0.001) and even more acidic with WT 750 (3.11, *P* < 0.001), as compared to that of PBS-treated mice. Both Y17H 750 and WT 750 differed from Y17H 375K. Like the nasal pH values, Y17H 750 increased cytosolic pH to 6.01 and 7.36 at 7 and 10 DPI, respectively. WT 750 and Y17H 375K cytosolic pH remained acidic at 7 DPI but was below the limit of detection at 10 DPI (Fig 7). The cytosolic pH in the tracheal epithelial cells was similar to the nasal cytosolic pH for all IAVs except for WT 750 at 5 DPI, which was acidic and remained acidic until 10 DPI in which no live cells were detected. Both Y17H 750 and Y17H 375K slightly decreased the mean pH from 5 to 10 DPI (Fig 8).

The late endosome and lysosomal baseline pH were acidic, with a mean of 5.03 for the late endosomes and 4.47 for the lysosomes in the nasal epithelium, 5.23 for the late endosomes and 3.90 for the lysosomes in the soft palate epithelium, 5.53 for the late endosomes and 3.51 for the lysosomes in the tracheal epithelium (Figs 6–8). The late endosome mean pH in the nasal epithelium increased to 6.6 (*P* < 0.5) with Y17H 750, to 5.7 (*P* < 0.5) with WT 750, and to 6.16 (*P* < 0.5) with Y17H 375K at 2 DPI. The late endosome mean pH then decreased at 5 DPI and differed between the Y17H and WT IAVs. Y17H 375 and WT 750 continued to decrease the mean pH until 10 DPI, whereas the mean pH with Y17H 750 recovered. The late endosome pH in the soft palate and tracheal epithelium exhibited the same degree of changes as that of the nasal epithelium for all IAVs except for Y17H 750, which showed the highest mean pH mean at 7 DPI and then returned to within 0.5 of the baseline pH. The mean lysosomal pH changed in the same manner as that of the late endosome but with an overall lower mean pH (Figs 6–8).

The secretory vesicles and microtubules were acidic at baseline in nasal, soft palate, and tracheal tissues, with a mean pH of 4.0–4.9 for the secretory vesicles and 4.80–5.2 for microtubules. The secretory vesicle and microtubule mean pH increased in the mean pH at 2 DPI, which was significantly increased in the nasal microtubule mean pH with both Y17H IAV inocula (*P* < 0.01 for Y17H 750 and *P* < 0.5 for Y17H375K), as compared to that of PBS-treated mice. WT 750 and Y17H375K decreased the microtubule mean pH until 10 DPI, whereas Y17H 750 recovered the mean baseline pH at 5 DPI and slightly increased at 7 and 10 DPI. In the soft palate epithelium, the microtubule mean pH slightly increased with Y17H 750 (5.15, *P* < 0.01) and Y17H 375K (5.28, P < 0.05 as compared with Y17H 750 and WT 750), with the highest increase in WT 750–infected mice (6.5, *P* < 0.001) at 2 DPI. At 5 DPI, Y17H 750 recovered the microtubule mean pH to that of the baseline, which increased by 0.5 at 7 DPI and by 0.2 at 10 DPI. WT 750 and Y17H 375K continued to decrease the mean microtubule pH and the number of live cells during infection (Fig 7).

Although the changes in the mean pH of the nasal and tracheal secretory vesicles did not differ among IAVs or during infection, the soft palate secretory vesicle pH significantly changed during infection with all IAVs similar to the changes in microtubule pH (Fig 7). The microtubule pH in the tracheal epithelium exhibited the same degree of change as that of the nasal epithelium but without significant differences between IAVs (Fig 8). Overall, the intracellular pH markedly increased in the subcellular compartment at 2 DPI, followed by a decrease during infection. By 7 DPI, the subcellular compartment pH of mice infected with Y17H IAVs returned to baseline, whereas mice infected with WT IAV had reduced populations of viable cells (Figs 6–8)

## Discussion

In this study, pH1N1 infection in mice and its effect on extracellular and intracellular respiratory tract pH were investigated. Infection with 750 PFU of WT virus, which resulted in approximately 20% mortality and 15% mean maximum weight loss, yielded peak viral loads and lymphocyte infiltration into nasal, soft palate, and tracheal tissues within the first 2–5 days of infection. Prior to infection, extracellular pH was approximately 7.8 in the upper respiratory tract. Infection with pH1N1 resulted in mild acidification with maximal reductions in pH at 5 DPI to the following pH values: naris 5.9, nasal sinus 6.2, soft palate 6.5, and trachea 6.5. Intracellularly, infection also reduced the pH of early and late endosomes. WT virus had an HA protein with an activation pH of 5.5, which has been considered near optimal for infection in mice [39], and has been shown to be resistant to extracellular inactivation by pH 6.4 media [38]. Mutant virus HA1-Y17H had a destabilized HA protein that is activated at pH 6.0, and this virus has been shown to susceptible to inactivation in pH 6.4 media [38]. Replication and host responses in mice infected with 750 PFU Y17H were highly attenuated. Y17H caused no weight loss or mortality, and viral titers were reduced compared to WT approximately 100-fold in nasal, soft palate, and tracheal tissues. 750-PFU infection with Y17H stimulated local lymphocyte infiltration and was previously shown to enhance type I interferon responses in dendritic cells [38]. Attenuated infection resulted in dampened extracellular acidification with maximal reductions in pH as follows: naris 6.8, nasal sinus 6.8, soft palate 6.5, and trachea 7.1. Y17H was previously shown to be susceptible to inactivation by pH 6.4 but not pH 7.0 media [38], thus its attenuation and resultant dampened acidification would be only expected to lead to feedback inactivation in the soft palate but not the naris, nasal sinus, or trachea. Inoculation of Y17H at a 500-fold higher dose (i.e., 375,000 PFU) increased weight loss, mortality, viral loads, and lymphocyte infiltration to levels comparable to infection with 750 PFU WT. Moreover, 375K infection with Y17H caused airway acidification as seen with WT with the following maximal reductions in extracellular pH: naris 6.5, nasal sinus 6.5, soft palate 6.4, and trachea 6.4. Under these mildly acidic conditions, Y17H is increasingly susceptible to inactivation [38]; therefore, an increase in Y17H dose to overcome attenuation triggered a negative feedback loop that would be expected to attenuate replication further than type I interferon responses described previously [38].

Overall, these studies demonstrate that pH1N1 infection in mice reduces respiratory pH to a level that may inactivate IAVs with destabilized HA proteins (activation pH approximately 6.0) but most likely not those of moderate stability (pH 5.5). Mild respiratory acidification in response to infection suggests a primal antiviral mechanism that may influence the evolution of respiratory pathogens. When inoculated intranasally in ferrets, Y17H-infected animals had reduced and delayed nasal titers and were incapable of transmitting by the airborne route; however, selection of a stabilized HA variant (H17H/R106K) that had an activation pH of 5.3 enabled airborne transmission [30]. In the background of A/England/195/2009 (H1N1), air-emitted Y17H virus produced significantly fewer plaques than those produced by E31K, which has an activation pH of 5.3 [40]. Circulating pH1N1 viruses isolated in 2009 had HA activation pH values of 5.5 [14,30,39], while those circulating from 2010–2012 had acquired one or more stabilizing mutations that reduced the HA activation pH to 5.2–5.4 [30,35–37]. Thus, mutant viruses with relatively unstable HA proteins have been shown to have fitness disadvantages in ferrets and humans.

In vivo fiber-optic pH sensing has been used previously in dogs [46], mice [47], swine [48], and humans [49,50]. This is the first study to our knowledge to measure extracellular pH in the respiratory tracts of pH1N1-infected animals. These studies were complemented with ex vivo single-cell analyses of the impact of infection on intracellular pH. In general, a homeostatic balance between the extracellular and intracellular pH is maintained, possibly to regulate changes in enzymatic activities and signal transduction [47]. Results from the present study suggest pH1N1 infection may shift this balance.

A previous study measuring the pH of cell surface viral-bound cells also showed variations in extracellular pH and reduced intracellular pH during IAV infection [51]. In this case, changes in pH were ascribed to protonation of M2 ion channels that scavenge protons from the acidic endosome during viral entry and budding, stimulating membrane fusion that enables release of the viral genome. M2 activation then initiates H^+^ release to the cytoplasm, which leads to decreased cytoplasmic pH [52–54]. High ATP consumption is linked to IAV replication and increased RNA synthesis [55,56]. In response to high ATP intake, infected cells synthesize ATP by oxidative metabolism and glycolysis [57]. This is followed by reversible conversion of pyruvate into lactate, resulting in a high rate of interaction between lactate and the H^+^ gradient across the plasma membrane in infected cells [58,59]. In agreement with these findings, we found that infection with both WT and Y17H IAVs changed the extracellular and intracellular pH during infection. The mechanism of altered pH has been identified here but has not yet been addressed. It is possible that H^+^ production in the cytoplasm leads to an increase in the extracellular environment near the cell membranes. High glycolysis levels and increased ATP production, which are needed for viral replication host cells, may also play a role. In other studies, glycolysis has been shown to produce more metabolic acid and H^+^ in the cytoplasm [58,59].

Our finding of acidic extracellular pH and high salinity at 2 and 5 DPI suggest that IAV infection leads to acidification of the respiratory tract, in which the activity of host ATPase [55] and ion levels are changed in infected cells [60,61]. Extracellular pH shifts are linked to ion and buffer exchanges on both sides of the cell membrane, and intracellular pH is not consistent in the cytoplasm [62,63]. Several cell membrane ion channels (e.g., Na+, Cl−, and K+) regulate cells by functioning as a volume-regulated anion through water and ion transfers [64]. H+ pump and Na+/H+ exchange in the plasma membrane control and regulate intracellular pH [65]. Increased calcium concentrations in response to pH modifications and membrane-permeant weak acids and bases are reported in different cell types [66,67]. Cytosolic alkalinization modulates epithelial fluid secretion and markedly increases cytosolic calcium by mobilization of calcium from internal storage compartments [26,66].

Our finding of reduced intracellular pH during IAV infection is supported by studies showing reduced intracellular pH during Sindbis virus infection [21]. This decreased intracellular pH may also occur after lytic infections with several other viruses, such as vesicular stomatitis virus [68], poliovirus [69], and herpes simplex virus [70], which alter different transport systems in the plasma membrane. Similarly, decreased intracellular pH after IAV infection in the present study is most likely due to protonation of the M2 ion channel, which is needed for IAV entry and budding. Furthermore, M2 balances the pH in the lumen of the trans-Golgi network with that of the cytoplasm, thereby inhibiting premature conformational changes in the viral HA during egress [71]. This may increase the pH inside the trans-Golgi network and decrease the pH in the cytoplasm and may be driven by increasing H^+^ export from the intracellular compartment [72].

Local innate immune cells in the respiratory tract are a critical first line of protection against IAV infections. The intracellular and extracellular acidic pH of infected cells may affect the function of local immune cells during IAV infection by activating multiple cell-signaling cascades (e.g., antiviral production, cytokines release, and programmed cell death) [73]. Our finding that the intracellular pH increased at 2 DPI in both groups may be a result of the stimulation of cytokine secretion from the airway epithelium of the infected cells, which attracts local immune cells (e.g., neutrophils, macrophages, monocytes, DCs, natural killer cells, and lymphocytes) to the infection site. These cells are activated to combat the viral infection and protect the airway epithelium and trigger the adaptive immune response [74]. The differential host responses against IAV infection result in resistant hosts that better recognition and clear infection, tolerant hosts with innate resistance that minimize infection-related immunopathology and tissue damage, and susceptible hosts with unmitigated infection or inadequate resistance to the negative consequences of immune responses to infection [75]. Such effects are displayed, in part, in mice infected with Y17H or WT at 7 and 10 DPI. At these times, stimulation of innate immune responses to infection by Y17H may help control viral replication, especially during the early stages of infection, while subsequent CD4 and CD8 activation help promote viral clearance and recovery [76]. The activation of DCs and alveolar macrophages in the Y17H group may also play important roles in clearing virus-infected cells by recruiting phagocytes, macrophages, and natural killer cells [77]. WT pH1N1 virus is a poorer inducer of innate immune responses [78,79].

In conclusion, our findings describe changes in extracellular and intracellular pH during pH1N1 infection in mice. IAVs are susceptible to inactivation by acidic environments according to the stabilities of their HA proteins. IAV infection in mice caused reductions in extracellular pH to a mildly acidic level sufficient to inactivate virions with HA activation pH values near 6.0. Viruses with HA proteins activated at pH 5.5 would be stable enough to avoid such inactivation by mild respiratory acidification. Further studies are needed to fully characterize mechanisms by which IAV infection alters respiratory pH and the impact of this phenomena on IAV evolution and interspecies adaptation.

## Supporting information

S1 File (XLSX)

S2 File (XLSX)

S3 File (XLSX)

S4 File (XLSX)

S5 File (XLSX)

S6 File (XLSX)

S7 File (XLSX)

S8 File (XLSX)

S9 File (XML)

S10 File (XML)
